# HDL enhances oxidation of LDL in vitro in both men and women

**DOI:** 10.1186/1476-511X-4-25

**Published:** 2005-10-20

**Authors:** T Solakivi, O Jaakkola, A Salomäki, N Peltonen, S Metso, T Lehtimäki, H Jokela, ST Nikkari

**Affiliations:** 1 Department of Medical Biochemistry, University of Tampere, Medical School, Tampere, Finland; 2 Institute of Medical Technology, University of Tampere, Tampere, Finland; 3 Laboratory of Atherosclerosis Genetics, Department of Clinical Chemistry, Tampere University Hospital, Tampere, Finland; 4 Department of Internal Medicine, Tampere University Hospital, Tampere, Finland

## Abstract

**Background:**

Oxidative modification of low-density lipoprotein (LDL) is a key event in the oxidation hypothesis of atherogenesis. Some *in vitro *experiments have previously suggested that high-density lipoprotein (HDL) co-incubated with LDL prevents Cu^2+^-induced oxidation of LDL, while some other studies have observed an opposite effect. To comprehensively clarify the role of HDL in this context, we isolated LDL, HDL_2 _and HDL_3 _from sera of 61 free-living individuals (33 women and 28 men).

**Results:**

When the isolated LDL was subjected to Cu^2+^-induced oxidation, both HDL_2 _and HDL_3 _particles increased the rate of appearance and the final concentration of conjugated dienes similarly in both genders. Oxidation rate was positively associated with polyunsaturated fatty acid content of the lipoproteins in that it was positively related to the content of linoleate and negatively related to oleate. More saturated fats thus protected the lipoproteins from damage.

**Conclusion:**

We conclude that *in vitro *HDL does not protect LDL from oxidation, but is in fact oxidized fastest of all lipoproteins due to its fatty acid composition, which is oxidation promoting.

## Background

Epidemiological studies show an inverse correlation between high-density lipoprotein (HDL) concentration and the risk of developing coronary artery disease [1]. According to a widely accepted hypothesis, HDL or its subtractions play an important role in recruiting and transporting cholesterol from peripheral tissues to the liver for excretion, a series of events known as reverse cholesterol transport [2]. Other properties of HDL link its antiatherogenic functions to its antioxidative effects. Some studies have shown that co incubation of LDL with HDL in the presence of divalent copper prevents the oxidative modification of LDL [3]. In some reports this finding could not be confirmed, and in fact it has been demonstrated that *in vitro *HDL is oxidized faster than other lipoproteins [4]. When HDL is oxidatively modified, it alters to a form that causes macrophages to accumulate cholesterol. [5]. It has been suggested that systemic inflammation gives rise to prooxidant and proinflammatory HDL particles [6]. Oxidatively modified HDL is found in atheromatous plaques from human aorta [7]. Oxidatively modified HDL is no longer capable of removing cholesterol from cells, and it enhances LDL oxidation [8].

The contradictory findings on the role of HDL on LDL oxidation in vitro may be due to rather small study populations, and the reported heterogeneity of oxidation kinetics between lipoprotein preparations *in vitro *[9] which might be due to individual intrinsic properties of the lipoproteins. In the present paper we report the results of a study of the effect of HDL subtractions and gender on *ex vivo *oxidation of LDL from a population of 61 healthy free-living human subjects.

## Results

Background characteristics of the men and women participating in the study are shown in Table 1. Compared with women, men had higher body mass indices, serum total cholesterol as well as LDL and apoB concentrations. Men had smaller LDL size and smaller concentrations of serum HDL and apoA-I than women.

**Table 1:** Characteristics of the study subjects.

	Women	Men	All
N	32	27	59
Age (years)	39.3 ± 10.5	39.3 ± 11.0	39.3 ± 10.6
Body mass index (kg/m^2^)	23.2 ± 3.1	25.8 ± 3.6**	24.4 ± 3.6
Total cholesterol (mmol/l)	5.23 ± 0.87	5.76 ± 1.02*	5.47 ± 0.97
Triacylglycerol (mmol/l)	0.96 ± 0.41	1.81 ± 1.27***	1.35 ± 0.71
HDL cholesterol (mmol/l)	1.88 ± 0.32	1.38 ± 0.23***	1.65 ± 0.38
LDL cholesterol (mmol/l)	2.91 ± 0.84	3.63 ± 0.92**	3.21 ± 0.96
ApoA-I (g/l)	1.70 ± 0.20	1.46 ± 0.13***	1.59 ± 0.21
ApoB (g/l)	0.81 ± 0.20	1.00 ± 0.22**	0.90 ± 0.23
Lp(a) (U/l)	266 ± 226	165 ± 201	220 ± 219
fB-Glucose (mmol/l)^a^	4.4 ± 0.4	4.6 ± 0.6	4.5 ± 0.5
LDL diameter (nm)	26.94 ± 0.45	26.40 ± 0.55***	26.70 ± 0.51

Values are mean ± SD. ^a ^fB, fasting blood, * p < 0.05, ** p < 0.01, *** p < 0.001 compared to women by Mann-Whitney U-test.

The fatty acid compositions of ultracentrifugally isolated LDL, HDL_2 _and HDL_3 _fractions were analyzed by gas liquid chromatography (Table 2). There were no gender differences in the total amounts of saturated, monounsaturated and polyunsaturated fatty acids of LDL, HDL_2 _and HDL_3_. The calculated peroxidizability indices were also similar in men and women. In both genders this index increased significantly from LDL to HDL_2 _to HDL_3 _(p < 0.001, Wilcoxon's matched pairs test).

**Table 2:** Percentage composition of fatty acids of LDL, HDL_2 _and HDL_3 _in 59 healthy subjects.

		Women			Men	
Fatty acid	LDL	HDL_2_	HDL_3_	LDL	HDL_2_	HDL_3_

14:0	0.80 ± 0.22	0.67 ± 0.19	0.61 ± 0.17	0.97 ± 0.29*	0.94 ± 1.41***	0.76 ± 0.26*
16:0	19.50 ± 1.27	22.68 ± 1.41	22.45 ± 1.51	19.39 ± 1.23	22.79 ± 1.30	22.25 ± 1.10
16:1(n-7)	2.28 ± 0.64	1.72 ± 0.51	1.69 ± 0.51	2.22 ± 0.79	1.82 ± 0.71	1.87 ± 1.21
18:0	5.44 ± 0.58	8.18 ± 0.91	8.24 ± 0.90	5.71 ± 0.35	8.41 ± 0.67	8.71 ± 0.60
18:1T	0.35 ± 0.11	0.38 ± 0.12	0.36 ± 0.12	0.38 ± 0.15	0.45 ± 0.19	0.42 ± 0.16
18:1(n-9)	21.44 ± 1.63	17.79 ± 1.21	16.91 ± 1.02	21.12 ± 2.37	19.62 ± 3.09	17.77 ± 2.29
18:2(n-6)	34.92 ± 2.88	30.20 ± 2.93	30.71 ± 3.00	34.92 ± 4.16	28.38 ± 3.90	29.41 ± 3.72
18:3(n-3)	0.87 ± 0.22	0.67 ± 0.18	0.65 ± 0.18	0.87 ± 0.20	0.77 ± 0.23	0.69 ± 0.18
18:3(n-6)	0.43 ± 0.14	0.28 ± 0.09	0.28 ± 0.01	0.53 ± 0.26	0.34 ± 0.20	0.35 ± 0.20
20:0	0.31 ± 0.04	0.26 ± 0.03	0.22 ± 0.03	0.26 ± 0.04***	0.23 ± 0.03***	0.19 ± 0.02**
20:3(n-6)	1.22 ± 0.29	1.85 ± 0.44	1.97 ± 0.49	1.34 ± 0.26	1.86 ± 0.39	2.09 ± 0.40
20:4(n-6)	5.40 ± 0.91	7.08 ± 1.04	7.70 ± 1.20	5.53 ± 1.14	6.67 ± 1.42	7.64 ± 1.49
20:5(n-3)	1.12 ± 0.59	1.28 ± 0.67	1.38 ± 0.75	1.26 ± 0.59	1.33 ± 0.55	1.50 ± 0.59
22:0	0.91 ± 0.10	0.75 ± 0.13	0.63 ± 0.14	0.86 ± 0.11	0.60 ± 0.12	0.55 ± 0.13
22:5(n-3)	0.40 ± 0.11	0.65 ± 0.16	0.68 ± 0.16	0.47 ± 0.07**	0.74 ± 0.12*	0.82 ± 0.11**
22:6(n-3)	2.18 ± 0.50	3.46 ± 0.73	3.70 ± 0.79	1.96 ± 0.53	3.25 ± 0.86	3.37 ± 0.88
24:0	0.84 ± 0.07	0.65 ± 0.09	0.56 ± 0.08	0.83 ± 0.14	0.63 ± 0.13	0.56 ± 0.12
24:1(n-9)	1.59 ± 0.21	1.43 ± 0.23	1.23 ± 0.21	1.39 ± 0.28**	1.17 ± 0.24**	1.04 ± 0.19*
ΣSAFA	27.82 ± 1.21	33.21 ± 1.16	32.77 ± 1.23	27.88 ± 1.18	33.54 ± 1.50	32.94 ± 1.12
ΣMUFA	25.33 ± 2.03	20.98 ± 1.54	19.86 ± 1.34	24.48 ± 2.67	22.28 ± 3.16	20.51 ± 2.63
ΣPUFA	46.51 ± 2.55	45.44 ± 2.00	47.02 ± 1.82	47.26 ± 3.66	43.75 ± 4.13	46.14 ± 3.48
PI	83.2 ± 7.4	96.1 ± 8.3	101.2 ± 9.3	83.7 ± 9.2	92.2 ± 12.0	99.2 ± 10.9

Values are mean ± SD. * p < 0.05. ** p < 0.01, *** p < 0.001 compared to women. ΣSAFA, sum of percentages of saturated fatty acids, ΣMUFA, sum of percentages of monounsaturated fatty acids, ΣPUFA, sum of percentages of polyunsaturated fatty acids, PI, peroxidizability index (see methods).

The oxidation of LDL of all subjects gave rise to typical conjugated diene vs. time -curves, where the different phases of hydro peroxide formation were clearly discernible. Co incubations of LDL with either HDL_2 _or HDL_3 _produced biphasic profiles with faster oxidation in the beginning, followed by a slower rate and finally a faster propagation phase. The profiles looked similar in all participants.

Co incubation of LDL with HDL_2 _or HDL_3 _decreased the mean lag time of diene formation in both women and men (Table 3). Likewise, the mean propagation rate and the maximum diene concentration increased significantly in the presence of HDL. These oxidation parameters did not differ between women and men.

**Table 3:** Kinetic parameters of LDL, LDL + HDL_2 _and LDL + HDL_3 _oxidation in 59 healthy subjects.

	**Women**	**Men**	**All**
**LDL**			
Lag time (min)	60.9 ± 7.9	59.3 ± 6.9	60.2 ± 7.4
Rate (μmol/l/min)^a^	0.509 ± 0.067	0.502 ± 0.067	0.506 ± 0.066
Max (nmol/mg)^b^	541 ± 41	537 ± 51	539 ± 45
**LDL + HDL_2_**			
Lag time (min)	56.0 ± 5.7***	56.0 ± 6.7***	56.0 ± 6.1***
Rate (μmol/l/min)^a^	0.687 ± 0.062***	0.654 ± 0.084***	0.671 ± 0.073***
Max (nmol/mg)^b^	774 ± 40***	745 ± 71***	762 ± 56***
**LDL + HDL_3_**			
Lag time (min)	55.8 ± 5.4***	54.8 ± 5.4***	55.3 ± 5.4***
Rate (μmol/l/min)^a^	0.616 ± 0.064***	0.607 ± 0.070***	0.612 ± 0.066***
Max (nmol/mg)^b^	687 ± 40***	674 ± 60***	681 ± 50***

Values are mean ± SD. ^a ^Rate means maximal formation rate of conjugated dienes during oxidation. Calculation of the diene concentration is based on ε = 29500 of the conjugated dienes ^b ^Max is the maximal amount of dienes produced per mg of LDL protein. *** p < 0.001 in comparison with LDL alone in Wilcoxon's matched pairs test.

Multiple forward stepwise regression analysis was performed to estimate the effect of lipids and factors related to lipoprotein metabolism on oxidation parameters. Predictors for the multivariate analysis were selected on the basis of initial correlation analyses using Spearman's correlation coefficients. The resulting models formed consistent patterns of predictors. The results of the models for the mixtures of LDL + HDL_2 _are shown in Table 4. The results were similar for mixtures of LDL and HDL_3_, and for LDL alone (not shown). In these incubations, an increase in lag time was related to fasting blood glucose concentration, and a decrease in lag time was related to the peroxidizability index. Oxidation rate was positively associated with PUFA content of the lipoproteins. Maximum concentration of dienes was positively related to the content of linoleate and to the ratio of LDL to apoB, and negatively related to oleate.

**Table 4:** Multivariate regression models of factors predicting oxidation parameters in mixtures of LDL and HDL_2_.

Dependent variable	Independent variable	Standardized regression coefficient β	p-Value	Total model
LDL + HDL_2_				
Lag time (min)	fB-Glucose	0.410	0.00037	R^2 ^= 0.296
	PI of HDL_2_	-0.351	0.00276	p < 0.00005
LDL + HDL_2_				
Oxidation rate (μmol/ml/min)	ΣPUFA of LDL	0.424	0.0325	R^2 ^= 0.57
	ΣPUFA of HDL_2_	0.352	0.0129	p < 0.00000
LDL + HDL_2_				
Maximum diene	HDL_2 _18:2n-6	0.429	0.00015	R^2 ^= 0.55
concentration (nmol/mg)	LDL 18:1n-9	-0.292	0.0071	p < 0.00000
	LDL/apoB	0.287	0.0033	

Abbreviations: PI, peroxidizability index; ΣPUFA, sum of percentages of polyunsaturated fatty acids; LDL/apoB, the ratio of LDL cholesterol to apoB concentration. Stepwise forward regression analysis

Although the mean lag time was shorter in the presence of HDL_2 _or HDL_3_, there were nine subjects who had longer lag time when HDL_2 _was co incubated with LDL. We analyzed, whether there were any differences between these nine subjects and the rest of the study group that could explain the increased lag time. These nine subjects had a significantly smaller peroxidizability index of HDL_2 _(88.0 ± 12.3 vs. 95.4 ± 9.5, p < 0.05) than the rest of the group. Their LDL lag time was slightly shorter (55.7 ± 4.7 vs. 61.0 ± 7.7 μmol/l/min, p < 0.05) and their fasting blood glucose concentration was lower (4.1 ± 0.5 vs. 4.5 ± 0.5 mmol/l, p < 0.05).

## Discussion

We found that co incubation of HDL_2 _or HDL_3 _with LDL in the presence of Cu^2+ ^resulted in shortening of the mean lag time and acceleration of the oxidation rate in comparison with that of incubation of LDL alone. If lag time or propagation rate are thought of as indices of oxidation resistance, this outcome contradicts the role of HDL as an antioxidant. Our findings are in line with the studies by Bowry et al. [4], Suzukawa et al. [10], Schnitzer et al. [11], Ohmura et al. [12] and Raveh et al. [9], who have come to the conclusion that HDL is more easily oxidized than LDL. In other studies, HDL has appeared to be less prone to oxidation and even to protect LDL against copper-induced oxidation [3,13-15]. In the study of Kontush et al. [16] all subtractions of HDL exhibited limited capacities to protect LDL at early stages of oxidation. At later phases, small dense HDL particles were the most potent inhibitors of LDL oxidation under mildly oxidative conditions. If strongly oxidative conditions were used [5 μmol/l Cu^2+^), none of the HDL subtractions offered any protection to LDL. The results were fairly similar whether the subspecies were isolated from serum or EDTA-plasma despite their widely differing paraoxonase activities suggesting that paraoxonase may have had a smaller role in the inhibition. In our study the HDL_2 _subtraction of the majority of the subjects had properties that enhanced the onset of propagation phase. However, in 9 participants this phase was delayed in the presence of a moderate concentration of Cu^2+ ^emphasizing that the individual variation of intrinsic characteristics of lipoproteins can not be overlooked.

In all, kinetic analyses by different investigators of the effects of HDL on copper-induced peroxidation of LDL are difficult to compare. (a) Firstly, the concentrations of LDL and HDL have been inconsistent and their expression has been variably based on protein, total lipid, total mass, phospholipid or cholesterol concentration, molar concentrations or particle numbers. The investigations into the kinetics of lipoprotein oxidation of Raveh et al. [9] and Ziouzenkova et al. [17] showed that the lag time and the propagation rate are dependent on LDL concentration. (b) Secondly, copper concentrations have also differed between the experiments. This has profound implications, since it has been shown in kinetic experiments that the lag time and the oxidation rate are correlated with the copper concentration until a saturating concentration is reached [9,16,18]. However, until more data are available, there is reason to think that the number of copper binding sites of lipoproteins is not constant but varies [18]. (c) Thirdly, the ratio of copper to lipoprotein has varied between studies. It has been found that the kinetic profile of LDL oxidation changes in response to copper concentration and that the familiar monophasic, auto accelerating profile is only obtained when the copper concentration is relatively high [17]. All of the compound kinetic curves in our study had a biphasic shape. The first phase of rapid oxidation in such a profile, whether observed with HDL or LDL, has been interpreted to occur via a tocopherol-mediated mechanism [9] where vitamin E acts as a prooxidant. Because the rate of oxidation is regulated by the ratio of bound copper/lipoprotein as outlined above, the addition of HDL to LDL should, theoretically, have lengthened the lag time and oxidation rate instead of shortening it, since HDL bound part of the copper. Obviously, many factors are involved in determining the outcome of this kind of experiment. Furthermore, it is apparent that ex vivo oxidization experiments with lipoproteins require standardization.

Earlier studies have shown that there are several intrinsic properties of lipoproteins that can affect their susceptibility to oxidation. Lipoprotein antioxidant content [19,20], fatty acid composition [21,22], presence of various enzymes [13] and LDL and HDL size [16,23] are among the factors that have been shown to have an impact on oxidation parameters, the former especially in supplementation studies. Also, long-term habitual diets with different fatty acid contents have been shown to influence LDL oxidation susceptibility [24]. We analyzed the fatty acid compositions of LDL, HDL_2 _and HDL_3 _particles. Fatty acids are highly intercorrelated, and therefore their use in multivariate analysis as predictors is problematic. We tried to overcome this difficulty by uniting the information in the fatty acid profiles into a single term – the peroxidizability index – which describes the combined reactivity of fatty acids towards reactive oxygen species [25]. The results (Table 4) show that this index was significantly larger in both HDL_2 _and HDL_3 _than in LDL in men as well as in women, suggesting that HDL particles might be more susceptible to oxidation than LDL. This opinion was further strengthened by the findings that in multiple regression analysis the peroxidizability index of LDL, HDL_2 _or HDL_3 _in combination with fasting blood glucose concentration were the best predictors of lag time when LDL was oxidized alone or in mixtures with HDL_2 _or HDL_3_, respectively. Furthermore, our finding of a smaller peroxidizability index in those subjects whose lag time lengthened in the presence of HDL2 is in line with the suggestion that the rate of oxidation is governed by the ratio of bound copper to oxidizable lipids [26]. The results of our experiments also confirm the findings of earlier studies and suggest that the proportions of polyunsaturated fatty acids as well as those of linoleic acid and oleic acid [27-29] are related to the oxidation rate and the amount of dienes formed during in vitro oxidation. We have no ready explanation as to why the glucose concentration had a positive correlation with lag time of oxidation especially since all our subjects were normoglycemic. It has been shown that LDL isolated from patients with poorly controlled type I diabetes is more susceptible to copper-induced oxidation than LDL from control subjects [30]. Consequently, it has been suggested that glycated LDL might be particularly prone to oxidation. Nonetheless, our result is more in line with the results obtained in well-controlled type I diabetics, where glycated LDL gave a longer lag time than that of nonglycated LDL [31].

## Conclusion

In conclusion, we report that the lag time, the maximum propagation rate for the formation of dienes and the amount of dienes formed by Cu^2+ ^-induced oxidation of LDL alone or in the presence of HDL_2 _or HDL_3 _do not differ between healthy men and women despite significant differences in lipid concentrations. Our findings do not support the concept that co incubation of LDL with HDL in the presence of divalent copper prevents its oxidative modification. Rather, our findings support previous results that in vitro HDL is oxidized fastest of all lipoproteins [4] partly because of its fatty acid composition, which is oxidation promoting.

## Methods

### Subjects

61 healthy subjects from the personnel and medical students of the Department of Medical Sciences of Tampere University and Tampere University Hospital volunteered. The age range of the subjects was 20 to 58 years. 33 were women and 28 were men. All participants filled in a questionnaire, where emphasis was given to their health status (diseases and use of medication) in addition to health related behavior (smoking, use of alcohol and vitamins). Ten subjects were current smokers and two abstained from alcohol. Fasting blood glucose concentration was ≤5.7 mmol/l in all subjects. Nine women and two men reported the use of vitamins and 12 women used hormone preparations. The results of two of the participants were later removed from analysis because of reported diseases Thus, 59 subjects remained. All participants gave their written consent to the study. The study protocol was approved by the ethics committee of the Tampere University Hospital.

### Blood Samples

Fasting (12 h) blood samples were taken into suitable tubes (Vacuette, Greiner) from the antecubital vein in a sitting position after a 15-min rest using minimal stasis. Samples for the isolation of lipoproteins and for LDL size determination were taken into pre-chilled EDTA tubes, which were immediately placed in ice. Plasma was separated after centrifugation (Heraeus, 2000 xg, +4°C). EDTA plasmas were supplemented with sucrose (0.6 % w/v final concentration). This procedure has been shown to preserve LDL from oxidation for at least two months and the oxidation curve does not differ from that of a fresh sample [32]. All samples were kept frozen at -70°C until analyzed. Fasting blood glucose concentration was determined from capillary blood using Hemocue Glucose Analyzer (Hemocue, Ängelholm Sweden).

### Analysis of Lipids and Lipoproteins

Cholesterol, HDL cholesterol, triacylglycerol, apoA-I and apoB concentrations were measured with Cobas Integra 700 automatic analyzer (Roche Diagnostics, Basel, Switzerland) using reagents and calibrators as recommended by the manufacturer. LDL cholesterol was calculated according to Friedewald. Lp(a) concentrations were analyzed by radioimmunoassay (Pharmacia, Uppsala Sweden) according to the manufacturer's instructions.

### Isolation of Lipoproteins

Lipoproteins were fractionated by isopycnic density gradient ultracentrifugation using a Beckman SW40 Ti rotor in a Beckman L60 centrifuge (36000 rpm, 40 hours, 10°C). 2.0 ml of plasma was gently mixed with 4.0 ml of d 1.35 g/l NaCl-KBr solution in a polyallomer 14 × 95 mm tube. The mixture was then successively over layered with 4.5 ml of a d 1.006 salt solution and 1.0 ml of distilled water. The gradients were fractionated as described [33] and 0.4-ml fractions were collected. The fractions belonging to LDL, HDL_2 _and HDL_3 _were pooled on the basis of the absorbance curve. A part of the pooled fractions were immediately frozen to -70°C and a part was used for the oxidation experiments.

### Oxidation of Lipoproteins

The susceptibility of LDL and mixtures of LDL and HDL subtractions to in vitro copper-catalyzed oxidation was assessed by the technique described in [34] as modified from Esterbauer et al. [35]. LDL (50 μg protein/ml ≈ 0.1 μM) was incubated either alone or mixed with autologous HDL_2 _(50 μg protein/ml ≈ 0.35 μM) or HDL_3 _(50 μg protein/ml ≈ 0.53 μM). The protein concentrations were determined using the method of Markwell et al. [36]. Oxidation was started by adding 10 μl of CuSO4 to a final concentration of 1.65 μM Cu^2+^. The spectrophotometer was computer-operated (UVWinlab 2.1). This program also collected the absorbance data at 2-min intervals during the oxidation. Several characteristic indices were obtained from the resulting absorbance versus time curves [32]. To control the in vitro oxidation procedure we prepared an LDL pool as described [37] and stored it at -70°C in 0.15-M NaCl/1 mM EDTA solution containing 0.6 % sucrose. One control LDL was analyzed in every oxidation run. The inter-assay coefficient of variation for lag time was 3.1 %. This LDL preparation was also used as a standard in gradient gel electrophoresis.

### Electrophoretic Analysis of Lipoprotein Size

For the estimation of lipoprotein particle size in EDTA-plasma samples we used the nondenaturing gradient gel electrophoretic method of Krauss and Burke [38]. However, the 2–16 % polyacrylamide gels were cast in-house according to the instructions given by Pharmacia (Uppsala, Sweden) as described [39]. A control plasma sample (peak particle diameter 27.00 nm) stored at -70°C was included in every gel. The inter-assay coefficient of variation during this study was 1.1 %.

### Fatty Acid Composition of Lipoproteins

The fatty acid compositions of the ultracentrifugally isolated LDL, HDL_2 _and HDL_3 _fractions were analyzed by capillary gas-liquid chromatography. Lipids were extracted with chloroform/methanol, partitioned, and the chloroform phase was dried under N_2 _[40]. The lipids were then transesterified with H_2_SO_4 _in dry methanol at 85°C for 2 h under N_2_. Following the addition of water, methyl esters of the fatty acids were extracted with petroleum ether and analyzed in a Shimadzu GC-14A gas chromatograph (Shimadzu Corporation, Kyoto, Japan) with a flame ionization detector using a Supelco SP 2560 capillary column (100 m, 0.25 mm I.D., 0.20 μm film thickness). The carrier gas was helium. The column temperature was held at 180°C for 15 min and thereafter programmed to increase at 3°C/min until 230°C and held at this temperature for 40 min. The individual fatty acids were identified with the aid of a standard mixture of methyl esters (Lipid standards 189-15 and 189-17, Sigma). The areas were measured with a Shimadzu C-R4A Chromatopac Integrator and the results expressed as percentages of the sum of all fatty acids from 14:0 to 24:1. As a control sample we used a pool of isolated HDL that was kept frozen at -70°C. The inter-assay coefficients of variation for the percentages of different fatty acids ranged from 0.3 to 4.4 %. From fatty acid compositions, the following indices were calculated: saturated fatty acids (SAFA) = Σ(%) of saturated fatty acids; monounsaturated fatty acids (MUFA) = Σ(%) of monoenoic fatty acids; polyunsaturated fatty acids (PUFA) = Σ(%) of polyunsaturated fatty acids; peroxidizability index (PI) = [(Σ mol% monoenoic FAs × 0.025) + (Σ mol% dienoic FAs × 1) + (Σ mol% trienoic FAs × 2) + (Σ mol% tetraenoic FAs × 4) + (Σ mol% pentaenoic FAs × 6) + (Σ mol% hexaenoic FAs × 8)] [25].

### Statistical Analysis

Results are expressed as means ± standard deviation. Plasma triacylglycerol and Lp(a) concentrations were used as their logarithms but reported as their original results. Comparisons were conducted by analysis of variance or covariance and Mann-Whitney U-test. For pair wise comparisons we used Wilcoxon's matched pairs test. Univariate associations between variables were analyzed using Spearman's correlation coefficients. Predictors for the multivariate analysis were selected on the basis of the correlation analyses. Multivariate analysis was done using the stepwise forward linear regression technique. The Statistica for Windows (version 5.1) software package (Statsoft Inc., Oklahoma, USA) was used for the statistical analyses.

## List of abbreviations

HDL, high density lipoprotein; LDL, low density lipoprotein; SAFA, saturated fatty acids; MUFA, monounsaturated fatty acids;, PUFA, polyunsaturated fatty acids; PI, peroxidizability index

## Authors' contributions

TS and OJ conceived of the study, participated in its design, performed the statistical analysis and drafted the manuscript, NP and AS carried out the laboratory analyses, MS planned and analyzed the health questionnaire, TL, HJ and STN participated in the coordination of the study and helped to draft the manuscript. All authors read and approved the final manuscript.
